# Dichlorido[2-(pyridin-2-yl)-*N*-(pyridin-2-yl­methyl­idene)ethanamine-κ^3^ *N*,*N*′,*N*′′]manganese(II) monohydrate

**DOI:** 10.1107/S1600536812037877

**Published:** 2012-09-08

**Authors:** Daniel Tinguiano, Ibrahima Elhadj Thiam, Moussa Dieng, Mohamed Gaye, Pascal Retailleau

**Affiliations:** a Département de Chimie, Faculté des Sciences et Techniques, Université Cheikh Anta Diop, Dakar, Senegal; b Centre de Recherche de Gif sur Yvette, Institut de Chimie des Substances Naturelles, UPR2301-CNRS, 1 Avenue la Terrasse, 91198 Gif sur Yvette cédex, France

## Abstract

In the title complex, [MnCl_2_(C_13_H_13_N_3_)]·H_2_O, the Mn^II^ atom is in a distorted square-pyramidal environment, with an Addison τ parameter of 0.037. The coordination geometry is defined by three N-atom donors from the tridentate 2-(pyridin-2-yl)-*N*-(pyridin-2-yl­methyl­idene)ethanamine ligand and two terminal Cl atoms. Although the H atoms of the lattice water molecule were not located, O⋯O distances of 3.103 (7) Å and O⋯Cl distances of 3.240 (3) and 3.482 (4) Å suggest that hydrogen bonding is responsible for the stabilization of the crystal packing.

## Related literature

For the computation of the τ parameter describing the distortion of a square-pyramidal geometry, see: Addison *et al.* (1984 ▶). For a related structure, see: Marzec *et al.* (2011 ▶).
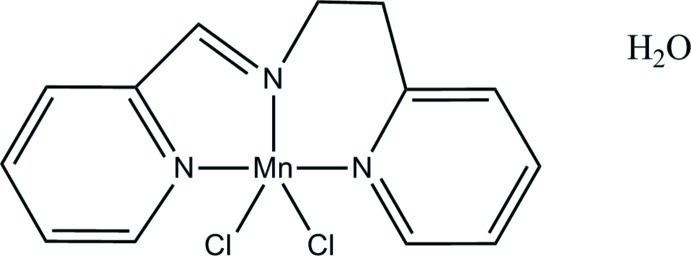


## Experimental

### 

#### Crystal data

[MnCl_2_(C_13_H_13_N_3_)]·H_2_O
*M* *_r_* = 355.12
Monoclinic, 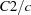

*a* = 19.173 (3) Å
*b* = 8.826 (1) Å
*c* = 18.088 (2) Å
β = 94.009 (2)°
*V* = 3053.4 (7) Å^3^
*Z* = 8
Mo *K*α radiation
μ = 1.21 mm^−1^
*T* = 293 K
0.26 × 0.24 × 0.20 mm

#### Data collection

Nonius KappaCCD diffractometer
Absorption correction: multi-scan [*SCALEPACK* in *CrystalClear-SM Expert* (Rigaku, 2009 ▶)] *T*_min_ = 0.69, *T*_max_ = 0.79
13865 measured reflections
2774 independent reflections
2039 reflections with *I* > 2σ(*I*)
*R*_int_ = 0.058

#### Refinement

*R*[*F* ^2^ > 2σ(*F* ^2^)] = 0.044
*wR*(*F* ^2^) = 0.113
*S* = 1.02
2773 reflections
181 parameters
H-atom parameters constrained
Δρ_max_ = 0.49 e Å^−3^
Δρ_min_ = −0.43 e Å^−3^

### 

Data collection: *CrystalClear-SM Expert* (Rigaku, 2009 ▶); cell refinement: *CrystalClear-SM Expert*; data reduction: *CrystalClear-SM Expert*; program(s) used to solve structure: *SHELXS97* (Sheldrick, 2008 ▶); program(s) used to refine structure: *SHELXL97* (Sheldrick, 2008 ▶) and *CRYSTALBUILDER* (Welter, 2006 ▶); molecular graphics: *PLATON* (Spek, 2009 ▶); software used to prepare material for publication: *SHELXL97*.

## Supplementary Material

Crystal structure: contains datablock(s) I, global. DOI: 10.1107/S1600536812037877/fk2065sup1.cif

Structure factors: contains datablock(s) I. DOI: 10.1107/S1600536812037877/fk2065Isup2.hkl

Additional supplementary materials:  crystallographic information; 3D view; checkCIF report

## Figures and Tables

**Fig. 1.**
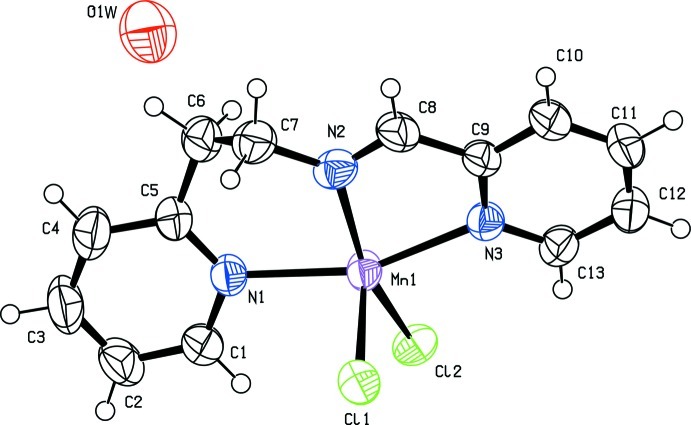
Molecular structure of the title compound with anisotropic displacement ellipsoids at the 50% probability level.

## supplementary crystallographic information

### Comment

In the title compound, the Mn^II^ ion displays a fivefold coordination geometry by three nitrogen atoms from the ligand molecule and two chloride atoms in terminal positions. A non-coordinated solvent water molecule is present. The bond lengths between the N atoms and the metal ion vary between 2.226 (3) Å [Mn1—N2] and 2.259 (3) Å [Mn1—N1]. These values are comparable to the bond lengths in similar manganese complex [2.2227 (16)–2.2628 (16) Å] (Marzec *et al.*, 2011). The Mn—Cl bond distances are 2.4554 (11) Å for Mn1—Cl1 and 2.4338 (10) Å for Mn1—Cl2. The Cl1—Mn1—Cl2 measures 99.89 (4)° and the angles between the Mn^II^ ion and the coordinating N atoms located in the basal plane vary between 74.40 (11) ° [N2—Mn1—N3] and 159.28 (11)° [N3—Mn1—N1]. The largest angles around the Mn^II^ center are: *β*=N2—Mn—Cl2=161.52 (8)° and *α* = N1—Mn—N3=159.28 (11)°. Since the distortion value of the coordination polyhedron, *τ*=(*β*-*α*)/60, is evaluated by the two largest angles a in five-coordination geometry (Addison *et al.*, 1984), the value of *τ*=0.037 which can be compared with the ideal value of 1 for a trigonal-bipyramidal environment and 0 for a square-pyramidal environment, indicates a distorted square-pyramidal geometry around the Mn center with N1, N2, N3, and Cl2 in the plane. The apical position is occupied by Cl1. The configuration around C8 is assigned to be *E*, as the torsion angles N2—C8—C9—C10 and C7—N2—C8—C9 are 178.9 (3)° and -178.7 (3)°, respectively.

### Experimental

[(2-pyridyl)-*N*-(2-pyridylmethyl)ethanamine] (0.2133 g, 1 mmol) was dissolved in 20 ml of methanol. To the resulting solution, MnCl_2_.4H_2_O (0.1979 g, 1 mmol) was added. Immediate color change was observed. The mixture was stirred at room temperature during 2 h. The solution was filtered off and concentrated to tenth. Crystals that separated from the brown solution were filtered off and recrystallized in methanol. On standing for two weeks, suitable X-ray crystals were obtained. Yield: 65%. Anal. Calc. for [C_13_H_15_Cl_2_N_3_OMn] (%): C, 43.97; H, 4.26; N, 11.83. Found: C, 43.72; H, 4.80; N, 11.77. Selected IR data (cm^-1^, KBr pellet): 1635.

### Refinement

All H(C) atoms were located in difference maps. They were then treated as riding in geometrically idealized positions, with C—H = 0.93 (aryl), or 0.97 Å (CH_2_), and with *U*_iso_(H)=1.2 *U*_eq_(C). Water-H atoms could not be detected reliably. One low-resolution reflection (111) was omitted due to beamstop shading (*OMIT* instruction in *SHELX97*-*L*)).

### Crystal data

**Table:**

[MnCl_2_(C_13_H_13_N_3_)]·H_2_O	*F*(000) = 1448
*M**_r_* = 355.12	*D*_x_ = 1.545 Mg m^−^^3^
Monoclinic, *C*2/*c*	Mo *K*α radiation, λ = 0.71070 Å
Hall symbol: -C 2yc	Cell parameters from 4419 reflections
*a* = 19.173 (3) Å	θ = 0.4–25.4°
*b* = 8.826 (1) Å	µ = 1.21 mm^−^^1^
*c* = 18.088 (2) Å	*T* = 293 K
β = 94.009 (2)°	Block, brown
*V* = 3053.4 (7) Å^3^	0.26 × 0.24 × 0.20 mm
*Z* = 8	

### Data collection

**Table:**

Nonius KappaCCD diffractometer	2774 independent reflections
Radiation source: fine-focus sealed tube, Nonius KappaCCD	2039 reflections with *I* > 2σ(*I*)
Graphite monochromator	*R*_int_ = 0.058
Detector resolution: 9 pixels mm^-1^	θ_max_ = 25.3°, θ_min_ = 3.0°
phi and ω scans	*h* = −22→20
Absorption correction: multi-scan [*SCALEPACK* in *CrystalClear-SM Expert* (Rigaku, 2009)]	*k* = −10→10
*T*_min_ = 0.69, *T*_max_ = 0.79	*l* = −21→20
13865 measured reflections	

### Refinement

**Table:**

Refinement on *F*^2^	Primary atom site location: structure-invariant direct methods
Least-squares matrix: full	Secondary atom site location: difference Fourier map
*R*[*F*^2^ > 2σ(*F*^2^)] = 0.044	Hydrogen site location: difference Fourier map
*wR*(*F*^2^) = 0.113	H-atom parameters constrained
*S* = 1.02	*w* = 1/[σ^2^(*F*_o_^2^) + (0.0548*P*)^2^ + 3.8894*P*] where *P* = (*F*_o_^2^ + 2*F*_c_^2^)/3
2773 reflections	(Δ/σ)_max_ < 0.001
181 parameters	Δρ_max_ = 0.49 e Å^−^^3^
0 restraints	Δρ_min_ = −0.43 e Å^−^^3^

### Special details

**Table:**

Geometry. All e.s.d.'s (except the e.s.d. in the dihedral angle between two l.s. planes) are estimated using the full covariance matrix. The cell e.s.d.'s are taken into account individually in the estimation of e.s.d.'s in distances, angles and torsion angles; correlations between e.s.d.'s in cell parameters are only used when they are defined by crystal symmetry. An approximate (isotropic) treatment of cell e.s.d.'s is used for estimating e.s.d.'s involving l.s. planes.
Refinement. Refinement of *F*^2^ against ALL reflections. The weighted *R*-factor *wR* and goodness of fit *S* are based on *F*^2^, conventional *R*-factors *R* are based on *F*, with *F* set to zero for negative *F*^2^. The threshold expression of *F*^2^ > σ(*F*^2^) is used only for calculating *R*-factors(gt) *etc*. and is not relevant to the choice of reflections for refinement. *R*-factors based on *F*^2^ are statistically about twice as large as those based on *F*, and *R*- factors based on ALL data will be even larger.

### Fractional atomic coordinates and isotropic or equivalent isotropic displacement parameters (Å^2^)

**Table:**

	*x*	*y*	*z*	*U*_iso_*/*U*_eq_	
Mn1	0.08475 (2)	0.08118 (6)	0.45536 (3)	0.04179 (19)	
Cl1	0.17062 (4)	0.04014 (11)	0.36281 (5)	0.0530 (3)	
Cl2	0.01984 (4)	−0.15558 (10)	0.44268 (5)	0.0488 (3)	
N1	0.16088 (13)	0.0210 (3)	0.55189 (15)	0.0442 (7)	
N2	0.11199 (14)	0.3215 (3)	0.48114 (17)	0.0486 (7)	
N3	0.01099 (13)	0.2207 (3)	0.38141 (15)	0.0416 (7)	
C1	0.18382 (19)	−0.1219 (4)	0.5525 (2)	0.0543 (9)	
H1	0.1642	−0.1882	0.5169	0.065*	
C2	0.2348 (2)	−0.1764 (5)	0.6028 (3)	0.0664 (11)	
H2	0.2492	−0.2770	0.6018	0.080*	
C3	0.2634 (2)	−0.0776 (5)	0.6541 (3)	0.0781 (14)	
H3	0.2984	−0.1096	0.6888	0.094*	
C4	0.2405 (2)	0.0689 (5)	0.6543 (3)	0.0713 (12)	
H4	0.2597	0.1362	0.6896	0.086*	
C5	0.18906 (16)	0.1179 (4)	0.6027 (2)	0.0465 (8)	
C6	0.16231 (18)	0.2766 (4)	0.6053 (2)	0.0556 (10)	
H6A	0.1888	0.3293	0.6451	0.067*	
H6B	0.1141	0.2727	0.6182	0.067*	
C7	0.16495 (18)	0.3695 (5)	0.5364 (2)	0.0565 (10)	
H7A	0.1580	0.4754	0.5481	0.068*	
H7B	0.2107	0.3593	0.5171	0.068*	
C8	0.07445 (18)	0.4208 (4)	0.4444 (2)	0.0515 (9)	
H8	0.0822	0.5235	0.4529	0.062*	
C9	0.01888 (16)	0.3713 (4)	0.38865 (19)	0.0433 (8)	
C10	−0.02351 (19)	0.4727 (4)	0.3488 (2)	0.0554 (10)	
H10	−0.0168	0.5765	0.3546	0.066*	
C11	−0.0758 (2)	0.4180 (5)	0.3002 (2)	0.0597 (10)	
H11	−0.1053	0.4843	0.2731	0.072*	
C12	−0.08389 (19)	0.2653 (5)	0.2925 (2)	0.0572 (10)	
H12	−0.1187	0.2259	0.2597	0.069*	
C13	−0.03961 (17)	0.1700 (4)	0.33402 (19)	0.0476 (8)	
H13	−0.0455	0.0659	0.3286	0.057*	
O1W	0.08035 (19)	0.2122 (4)	0.76769 (19)	0.0981 (11)	

### Atomic displacement parameters (Å^2^)

**Table:**

	*U*^11^	*U*^22^	*U*^33^	*U*^12^	*U*^13^	*U*^23^
Mn1	0.0446 (3)	0.0303 (3)	0.0486 (3)	−0.0041 (2)	−0.0107 (2)	0.0010 (2)
Cl1	0.0494 (5)	0.0530 (6)	0.0562 (6)	0.0021 (4)	0.0006 (4)	0.0027 (4)
Cl2	0.0553 (5)	0.0368 (5)	0.0541 (5)	−0.0117 (4)	0.0031 (4)	−0.0055 (4)
N1	0.0427 (15)	0.0465 (18)	0.0425 (17)	0.0020 (13)	−0.0028 (12)	0.0024 (14)
N2	0.0428 (15)	0.0470 (19)	0.0566 (19)	−0.0089 (13)	0.0068 (13)	−0.0112 (15)
N3	0.0437 (15)	0.0407 (17)	0.0400 (16)	0.0006 (12)	−0.0002 (12)	0.0004 (13)
C1	0.059 (2)	0.045 (2)	0.057 (2)	0.0075 (17)	−0.0052 (17)	−0.0016 (18)
C2	0.068 (2)	0.049 (3)	0.080 (3)	0.0153 (19)	−0.008 (2)	0.010 (2)
C3	0.072 (3)	0.073 (3)	0.084 (3)	0.010 (2)	−0.036 (2)	0.011 (3)
C4	0.067 (3)	0.073 (3)	0.070 (3)	−0.001 (2)	−0.027 (2)	−0.007 (2)
C5	0.0398 (18)	0.050 (2)	0.048 (2)	−0.0010 (15)	−0.0028 (15)	0.0024 (18)
C6	0.046 (2)	0.054 (2)	0.066 (3)	−0.0038 (16)	−0.0080 (17)	−0.006 (2)
C7	0.047 (2)	0.055 (2)	0.067 (3)	−0.0068 (17)	0.0008 (17)	−0.013 (2)
C8	0.055 (2)	0.035 (2)	0.064 (3)	0.0050 (15)	−0.0012 (18)	−0.0003 (18)
C9	0.0438 (18)	0.0358 (19)	0.050 (2)	0.0047 (14)	−0.0014 (15)	−0.0023 (16)
C10	0.060 (2)	0.042 (2)	0.062 (3)	0.0116 (17)	−0.0054 (18)	−0.0003 (19)
C11	0.057 (2)	0.067 (3)	0.053 (2)	0.0220 (19)	−0.0087 (17)	0.003 (2)
C12	0.049 (2)	0.071 (3)	0.050 (2)	0.0048 (18)	−0.0077 (17)	−0.005 (2)
C13	0.0491 (19)	0.047 (2)	0.046 (2)	−0.0033 (16)	−0.0023 (15)	−0.0049 (17)
O1W	0.115 (3)	0.091 (3)	0.087 (2)	0.012 (2)	−0.001 (2)	0.023 (2)

### Geometric parameters (Å, º)

**Table:**

Mn1—N2	2.226 (3)	C4—H4	0.9300
Mn1—N3	2.245 (3)	C5—C6	1.494 (5)
Mn1—N1	2.259 (3)	C6—C7	1.495 (5)
Mn1—Cl2	2.4348 (10)	C6—H6A	0.9700
Mn1—Cl1	2.4554 (11)	C6—H6B	0.9700
N1—C1	1.336 (5)	C7—H7A	0.9700
N1—C5	1.341 (4)	C7—H7B	0.9700
N2—C8	1.288 (5)	C8—C9	1.481 (5)
N2—C7	1.438 (4)	C8—H8	0.9300
N3—C13	1.327 (4)	C9—C10	1.378 (5)
N3—C9	1.343 (4)	C10—C11	1.373 (5)
C1—C2	1.376 (5)	C10—H10	0.9300
C1—H1	0.9300	C11—C12	1.363 (5)
C2—C3	1.360 (6)	C11—H11	0.9300
C2—H2	0.9300	C12—C13	1.380 (5)
C3—C4	1.366 (6)	C12—H12	0.9300
C3—H3	0.9300	C13—H13	0.9300
C4—C5	1.379 (5)		
			
N2—Mn1—N3	74.40 (11)	N1—C5—C6	119.8 (3)
N2—Mn1—N1	86.14 (11)	C4—C5—C6	120.3 (3)
N3—Mn1—N1	159.28 (11)	C5—C6—C7	117.2 (3)
N2—Mn1—Cl2	161.52 (8)	C5—C6—H6A	108.0
N3—Mn1—Cl2	96.78 (7)	C7—C6—H6A	108.0
N1—Mn1—Cl2	99.75 (8)	C5—C6—H6B	108.0
N2—Mn1—Cl1	97.16 (8)	C7—C6—H6B	108.0
N3—Mn1—Cl1	95.69 (7)	H6A—C6—H6B	107.2
N1—Mn1—Cl1	93.70 (7)	N2—C7—C6	110.8 (3)
Cl2—Mn1—Cl1	99.89 (4)	N2—C7—H7A	109.5
C1—N1—C5	118.7 (3)	C6—C7—H7A	109.5
C1—N1—Mn1	115.0 (2)	N2—C7—H7B	109.5
C5—N1—Mn1	126.0 (2)	C6—C7—H7B	109.5
C8—N2—C7	120.0 (3)	H7A—C7—H7B	108.1
C8—N2—Mn1	115.2 (2)	N2—C8—C9	120.0 (3)
C7—N2—Mn1	124.8 (3)	N2—C8—H8	120.0
C13—N3—C9	118.0 (3)	C9—C8—H8	120.0
C13—N3—Mn1	127.0 (2)	N3—C9—C10	122.2 (3)
C9—N3—Mn1	115.0 (2)	N3—C9—C8	115.4 (3)
N1—C1—C2	123.7 (4)	C10—C9—C8	122.3 (3)
N1—C1—H1	118.2	C11—C10—C9	118.9 (4)
C2—C1—H1	118.2	C11—C10—H10	120.5
C3—C2—C1	117.4 (4)	C9—C10—H10	120.5
C3—C2—H2	121.3	C12—C11—C10	119.1 (4)
C1—C2—H2	121.3	C12—C11—H11	120.5
C2—C3—C4	119.6 (4)	C10—C11—H11	120.5
C2—C3—H3	120.2	C11—C12—C13	119.1 (4)
C4—C3—H3	120.2	C11—C12—H12	120.5
C3—C4—C5	120.7 (4)	C13—C12—H12	120.5
C3—C4—H4	119.6	N3—C13—C12	122.7 (3)
C5—C4—H4	119.6	N3—C13—H13	118.6
N1—C5—C4	119.9 (4)	C12—C13—H13	118.6
			
N2—Mn1—N1—C1	−160.9 (3)	C2—C3—C4—C5	−0.6 (8)
N3—Mn1—N1—C1	179.2 (3)	C1—N1—C5—C4	−0.2 (5)
Cl2—Mn1—N1—C1	36.8 (2)	Mn1—N1—C5—C4	−173.2 (3)
Cl1—Mn1—N1—C1	−63.9 (2)	C1—N1—C5—C6	−177.5 (3)
N2—Mn1—N1—C5	12.3 (3)	Mn1—N1—C5—C6	9.5 (5)
N3—Mn1—N1—C5	−7.6 (5)	C3—C4—C5—N1	0.3 (7)
Cl2—Mn1—N1—C5	−150.0 (3)	C3—C4—C5—C6	177.6 (4)
Cl1—Mn1—N1—C5	109.3 (3)	N1—C5—C6—C7	−57.7 (4)
N3—Mn1—N2—C8	0.7 (2)	C4—C5—C6—C7	125.1 (4)
N1—Mn1—N2—C8	−172.1 (3)	C8—N2—C7—C6	135.2 (4)
Cl2—Mn1—N2—C8	−62.6 (4)	Mn1—N2—C7—C6	−42.0 (4)
Cl1—Mn1—N2—C8	94.7 (2)	C5—C6—C7—N2	73.8 (4)
N3—Mn1—N2—C7	178.0 (3)	C7—N2—C8—C9	−178.7 (3)
N1—Mn1—N2—C7	5.2 (3)	Mn1—N2—C8—C9	−1.2 (4)
Cl2—Mn1—N2—C7	114.7 (3)	C13—N3—C9—C10	0.0 (5)
Cl1—Mn1—N2—C7	−88.0 (3)	Mn1—N3—C9—C10	−178.2 (3)
N2—Mn1—N3—C13	−178.1 (3)	C13—N3—C9—C8	177.8 (3)
N1—Mn1—N3—C13	−157.4 (3)	Mn1—N3—C9—C8	−0.4 (4)
Cl2—Mn1—N3—C13	−14.7 (3)	N2—C8—C9—N3	1.1 (5)
Cl1—Mn1—N3—C13	86.0 (3)	N2—C8—C9—C10	178.9 (3)
N2—Mn1—N3—C9	−0.1 (2)	N3—C9—C10—C11	0.3 (5)
N1—Mn1—N3—C9	20.5 (4)	C8—C9—C10—C11	−177.3 (3)
Cl2—Mn1—N3—C9	163.3 (2)	C9—C10—C11—C12	−0.6 (6)
Cl1—Mn1—N3—C9	−96.0 (2)	C10—C11—C12—C13	0.5 (6)
C5—N1—C1—C2	0.4 (6)	C9—N3—C13—C12	−0.1 (5)
Mn1—N1—C1—C2	174.1 (3)	Mn1—N3—C13—C12	177.9 (3)
N1—C1—C2—C3	−0.6 (7)	C11—C12—C13—N3	−0.2 (6)
C1—C2—C3—C4	0.7 (7)		
